# Bradycardia From a Non-selective Beta-Adrenergic Antagonist, Timolol, Applied Ophthalmologically for Glaucoma

**DOI:** 10.7759/cureus.25815

**Published:** 2022-06-10

**Authors:** Gedaliah May, Daniel Miller, Daniel Fuchs

**Affiliations:** 1 Internal Medicine, Coney Island Hospital, Brooklyn, USA; 2 Internal Medicine, Icahn School of Medicine at Mount Sinai, Queens Hospital Center, Queens, USA

**Keywords:** drug-induced bradycardia, cardio, eye drops, general internal medicine, beta-blockers

## Abstract

Non-selective beta-adrenergic antagonists are used systemically to treat hypertension and tachycardia and are used ophthalmologically for glaucoma. Generally, ophthalmological medications don't have systemic effects, as they are applied specifically to a local area. In this case, however, it appears that timolol, a beta-blocker, had systemic effects on heart rate. This is something that prescribing physicians must be wary of when evaluating patients with glaucoma who have cardiovascular comorbidities. Additionally, patients should be informed of the importance of occluding the puncta when receiving eye treatments to lessen the risk of systemic effects.

## Introduction

Non-selective beta-adrenergic antagonists are generally used systemically to treat hypertension and tachycardia. Some beta-adrenergic antagonists are approved for use in glaucoma as well. It is rare to see systemic effects on blood pressure and heart rate when beta-adrenergic antagonists are used ophthalmologically. However, rarely, as in this case report, systemic effects can be seen with this route of administration. This can be due to the high vascularity that the medication can potentially access.

## Case presentation

**Figure 1:**
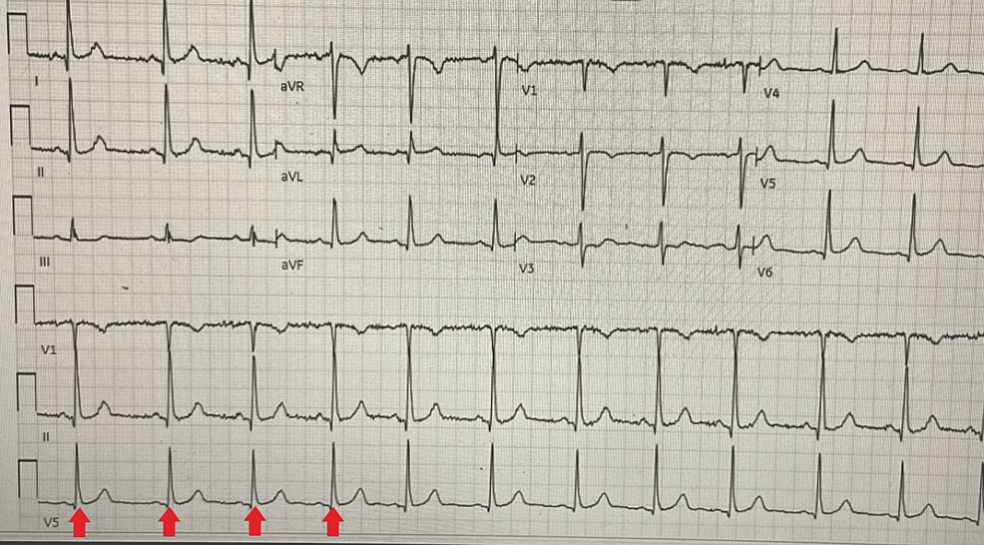
Baseline EKG Red arrows indicate the QRS complexes at a distance, measuring a heart rate of 69 beats per minute.

**Figure 2:**
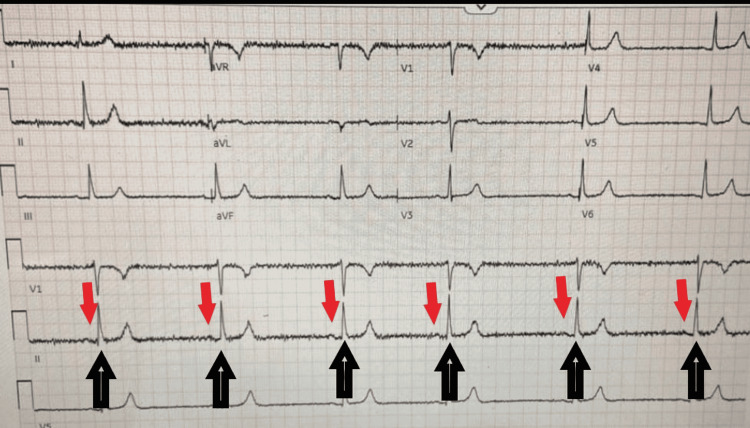
EKG showing sinus bradycardia Black arrows indicate the QRS complexes at a distance, measuring a heart rate of 36 beats per minute. Red arrows show the P waves, indicating that this is a sinus rhythm.

A 55-year-old female patient with a history of hypertension, glaucoma, colitis, sarcoidosis, obesity status post gastric sleeve seven years ago, and obstructive sleep apnea presented to the emergency department with complaints of shortness of breath, dizziness, chills, headache, and feelings of “passing out.” The patient denied any chest pain; she did, however, admit to lower abdominal non-radiating pain. The patient admitted to skipping her morning dose of amlodipine and hydrochlorothiazide. Vital signs revealed hypertension and bradycardia with a blood pressure of >200/>100 and a heart rate of 36. The patient had no past medical history of heart disease. EKGs from this patient over the past several years were reviewed and found to be unremarkable (Figure 1). Physical exam was unremarkable aside from hypertension and bradycardia with a normal S1 and S2. EKG (Figure 2) showed sinus bradycardia with prolonged Qtc. Complete blood count (CBC) and comprehensive metabolic panel (CMP) were unremarkable. Troponins were within normal limits. Thyroid-stimulating hormone (TSH) was slightly below the normal range but there was normal free T3 and free T4. The chest X-ray was unremarkable. CT abdomen and pelvis were unremarkable. Chest CT was unremarkable aside from a right thyroid lobe nodule. Thyroid ultrasound revealed multiple thyroid nodules. A non-contrast CT was performed and revealed an enlarged thyroid gland, which radiology reported to likely be a goiter. Timolol eye drops were discontinued, and she began to improve. A repeat EKG was performed, showing a normal sinus rhythm (Figure 2).

## Discussion

**Figure 3:**
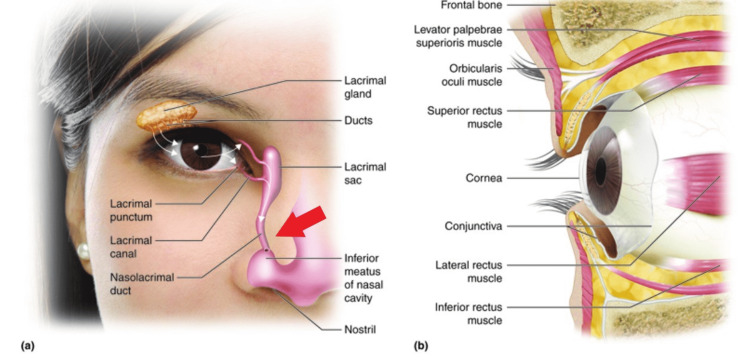
Anatomy of the nasolacrimal drainage system Anatomical features of the tissues surrounding the eye (a) and lacrimal system (b). The red arrow indicates where many of the vasculatures exist in the nasolacrimal drainage system, which is thought to be a conduit to systemic absorption. This work by Cenveo is licensed under a Creative Commons Attribution 3.0 United States (http://creativecommons.org/licenses/by/3.0/us/).

Timolol is a non-selective beta-adrenergic antagonist that is approved for use in glaucoma. The mechanism by which timolol works is by decreasing the ciliary epithelium’s production of aqueous humor, thereby decreasing intraocular pressure. Non-selective beta-blockers do have several side effects including but not limited to bradycardia. Although little literature is written on this, this applies to ophthalmological drops as well. It seems it is uncommon to have systemic effects from ophthalmological drops. One of the proposed mechanisms of systemic effects from ophthalmologically applied beta-adrenergic antagonists is absorption through the highly vascular nasal mucosa. Tears and other ocular liquids, such as ocular medications, drain from the eye by entering the puncta and traveling through the nasolacrimal duct, ultimately draining into the vascular nasal cavity at the inferior meatus (Figure 3) [1]. Furthermore, blinking of one's eye is thought to propel ocular fluids into the tear drainage system, therefore keeping the eye gently closed after ophthalmologic application can possibly help reduce systemic absorption [1-2]. Another possible solution to reduce the risk of systemic toxicity is to occlude the puncta while administering the medication as well as using gel ointments in place of the liquid formulation of the medication. Additionally, timolol is metabolized through the cytochrome P450 2D6 enzyme, therefore timolol poses a greater risk of cardiovascular side effects in poor cytochrome P450 2D6 metabolizers due to the higher circulating plasma concentration of the drug [3]. Furthermore, in patients who are poor cytochrome P450 2D6 metabolizers, the bradycardic effects may be seen more, especially during times of elevated physiologic beta-adrenergic stimulation (e.g. exercise) [3].

## Conclusions

Patients with glaucoma who present with bradycardia should be carefully evaluated for the use of beta-blocking ophthalmological drops. Additionally, potential alternatives to beta-adrenergic agents should be considered for glaucoma in any patient who is at increased risk of heart disease or bradycardia. Furthermore, gel formulations of ophthalmic timolol have been developed to reduce systemic absorption and adverse effects in comparison with conventional aqueous solution formulations.
